# Specialized Living Wound Dressing Based on the Self-Assembly Approach of Tissue Engineering

**DOI:** 10.3390/jfb9030053

**Published:** 2018-09-15

**Authors:** Laurence Cantin-Warren, Rina Guignard, Sergio Cortez Ghio, Danielle Larouche, François A. Auger, Lucie Germain

**Affiliations:** Centre de Recherche en Organogénèse Expérimentale de l’Université Laval/LOEX, Regenerative Medicine Division, CHU de Québec-Université Laval Research Centre, Département de Chirurgie, Faculté de Médecine, Université Laval, 1401 18e Rue, Québec, Québec G1J 1Z4, Canada; laurence.cantin-warren.1@ulaval.ca (L.C.-W.); rina.guignard@crchudequebec.ulaval.ca (R.G.); sergio.cortez-ghio.1@ulaval.ca (S.C.G.); danielle.larouche@crchudequebec.ulaval.ca (D.L.); francois.auger@fmed.ulaval.ca (F.A.A.)

**Keywords:** culture techniques, regenerative medicine, skin equivalent, tissue culture, bilayered skin substitutes, tissue engineering, skin ulcer

## Abstract

There is a high incidence of failure and recurrence for chronic skin wounds following conventional therapies. To promote healing, the use of skin substitutes containing living cells as wound dressings has been proposed. The aim of this study was to produce a scaffold-free cell-based bilayered tissue-engineered skin substitute (TES) containing living fibroblasts and keratinocytes suitable for use as wound dressing, while considering production time, handling effort during the manufacturing process, and stability of the final product. The self-assembly method, which relies on the ability of mesenchymal cells to secrete and organize connective tissue sheet sustaining keratinocyte growth, was used to produce TESs. Three fibroblast-seeding densities were tested to produce tissue sheets. At day 17, keratinocytes were added onto 1 or 3 (reference method) stacked tissue sheets. Four days later, TESs were subjected either to 4, 10, or 17 days of culture at the air–liquid interface (A/L). All resulting TESs were comparable in terms of their histological aspect, protein expression profile and contractile behavior in vitro. However, signs of extracellular matrix (ECM) digestion that progressed over culture time were noted in TESs produced with only one fibroblast-derived tissue sheet. With lower fibroblast density, the ECM of TESs was almost completely digested after 10 days A/L and lost histological integrity after grafting in athymic mice. Increasing the fibroblast seeding density 5 to 10 times solved this problem. We conclude that the proposed method allows for a 25-day production of a living TES, which retains its histological characteristics in vitro for at least two weeks.

## 1. Introduction

Chronic and non-healing wounds, including mixed and venous lower-extremity ulcers, are common health conditions that reduce patients’ quality of life and account for an important economic burden for the health care system [1,2,3]. Treatments with proven effectiveness for chronic venous leg ulcers include compressive products (bandages and stockings) and oral pentoxifylline [4]. However, these treatments are associated with high percentages of recurrence and healing failure [2,5]. Furthermore, large or lasting ulcers are known as being more difficult to manage [6,7,8]. In those circumstances, autograft or tissue flap harvested from an uninjured donor site on the patient may be applied to definitively close the wound. However, the harvesting procedure is painful, and can result in scarring, which can yield non-optimal aesthetical results [9,10].

Tissue-engineered skin substitutes containing living cells used as temporary dressings have been proven suitable to close wounds resistant to conventional therapies [11]. It has been demonstrated that living keratinocytes secrete soluble factors that trigger fibroblasts to release multiple wound-healing mediators [12]. When a skin substitute is composed of both living keratinocytes and fibroblasts, the secretion of various chemokines, cytokines, and growth factors, which are related to granulation tissue formation, angiogenesis, and epithelialization, is promoted [12,13,14,15]. This wound healing effect has been exploited in clinical studies where living cells were added into protein-based scaffolds, such as bovine collagen, polysaccharide-based biomaterials, decellularized native tissues [11,16,17,18], or synthetic biodegradable polymers or materials [19,20,21]. Scaffold-based methods offer the advantage of generating off-the-shelf and highly reproductible templates readily available for cell seeding. However, polymers and associated degradation products often elicit foreign body reactions [22,23], and vulnerability to bacterial infection of some polymeric or protein-based scaffolds represents a well-known challenge for clinicians and researchers [24]. Also, decellularization processes involve exposure to agents that cause extracellular matrix (ECM) modifications, leave cell debris or residual DNA which may contribute to adverse host responses [25].

The self-assembly approach developed at the LOEX laboratory [26] allows for the production of a scaffold-free cell-based tissue-engineered skin substitute (TES), including both a dermis and an epidermis [27]. This method is based on the capacity of mesenchymal cells, such as dermal fibroblasts, to produce their own ECM resulting in a manipulable tissue, which makes the use of scaffolds unnecessary. Autologous TES produced by the self-assembly approach, also known as the Self-Assembled Skin Substitute (SASS) [28], has led to successful permanent engraftment in severely burned patients [29], and was previously used to treat six patients with venous ulcers in a small clinical trial [30]. Low infection rate has been observed in these trials, and the SASS healed with few visible scars suggesting that foreign body reactions or prolonged inflammation have not occurred. However, the production of such cell-based skin substitutes is challenging due to the culture time required to naturally-secrete an ECM and the cell’s high inter-donor variability. 

We hypothesized that the 45-day standard method to produce SASS [28] can be adapted to produce a TES suitable for use as bioactive dressing more rapidly with fewer manipulations. Therefore, the aim of this study was to produce specialized living wound dressings based on the self-assembly approach of tissue engineering, while considering production time, handling effort during the manufacturing process and stability of the final product. Herein, we propose a method allowing for the production of a TES containing fibroblasts and keratinocytes within 25 days. The TES is stable for at least two weeks in an incubator.

## 2. Results

### 2.1. Macroscopic Appearance and Histological Analysis of Tissues Cultured in Vitro 

**Figure 1:**
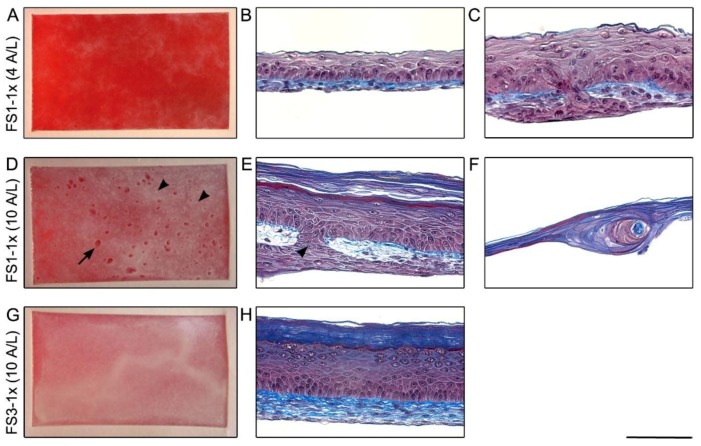
Macroscopic and histological analysis of tissue-engineered skin substitutes matured in vitro. Representative macroscopic pictures (left panel) and histological staining (right panel) of FS1-1× (**A**–**F**), and FS3-1× (**G**,**H**) cultured for 4 (4 A/L) and 10 (10 A/L) days at the air–liquid interface. **A**,**D**,**G** are top view pictures of the TES in their culture flasks, the red appearance is due to the culture media (red) that is under the TES. The arrowheads point to opaque white dots correlated with keratinocyte infiltrations across and under the reconstructed dermis (**E**) and arrow points a clear area correlated with ECM digestion zone (**F**). Note the whitish appearance of the FS3-1× (**G**) indicating a well differentiated epidermis (**H**). Histological coloration: Masson’s trichrome. Scale bar: **A**,**D**,**G**, 25 mm; **B**,**C**,**E**,**F**,**H**: 100 µm.

As a starting point, the self-assembly method to produce TES referred to as SASS-3 in a previous report [28] was chosen over the self-assembly approach originally presented by our team in 1999 [27] because a high quality tissue can be obtained in a shorter time, without using a material-based scaffold. This method involves the stacking of 3 fibroblast-derived tissue sheets (FSs) obtained by culturing 4 × 10^3^ fibroblasts/cm^2^ (seeding density herein referred to as 1×, see below for higher density [5× or 10×]) with ascorbic acid to reconstruct a dermis (herein referred to as FS3-1×). However, the stacking procedure is cumbersome and requires high technical skills. To bypass this critical step, the first attempt was to produce TES according to the method SASS-3, with a single FS to reconstruct the dermis (referred to as FS1-1×). In addition, the period of maturation at the air–liquid interface (A/L) was reduced to 4 days instead of 10. After 4 days of culture at the A/L, TESs composed of one FS (FS1-1×, Figure 1A) were less opaque compared with TESs composed of 3 FSs (FS3-1×, Figure 1G). After 10 days of maturation at the A/L, clearer zones were visible on the surface of FS1-1× (Figure 1D, arrow), while FS3-1× (Figure 1G) appeared macroscopically as a whitish tissue resembling normal human skin.

Histological cross-sections revealed that after 4 days of maturation at the A/L, the epidermal differentiation of FS1-1× was incomplete (Figure 1B). Indeed, basal keratinocytes were less organised in some areas and neither stratum granulosum nor stratum corneum were observed. Furthermore, keratinocyte infiltrations within the dermis were observed (Figure 1C). After 10 days of maturation at the A/L, opaque and homogeneous zones in FS1-1× were associated with more advanced epidermal differentiation (Figure 1E), while clearer areas correlated with ECM digestion zones (Figure 1F). Small opaque dots were also noted on the surface of FS1-1× after 10 days of maturation at the A/L (Figure 1D, arrowheads) and were associated with keratinocyte infiltrations across and under the dermis (Figure 1E, arrowhead). 

**Figure 2:**
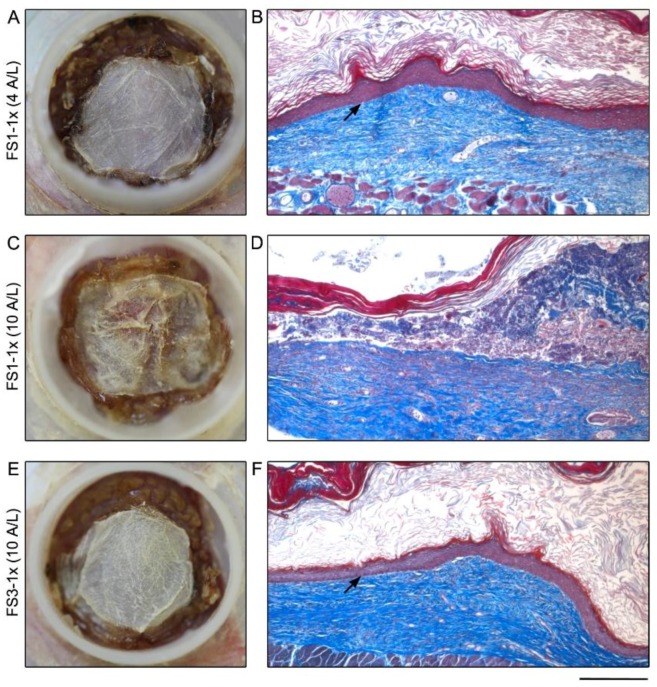
Macroscopic and histological analysis of tissue-engineered skin substitutes matured 21 days in vivo. Representative macroscopic (left panel) and histological (right panel) results of FS1-1× (**A**–**D**) and FS3-1× (**E**,**F**) cultured for 4 (4 A/L) and 10 (10 A/L) days at the air–liquid interface before grafting in athymic mice. Arrows point out the fully differentiated epidermis well attached to the underlying dermis. Note that when the air–liquid interface culture period of FS1-1× was prolonged to 10 days before grafting, epidermis was absent in several areas after in vivo maturation (**D**). Histological coloration: Masson’s trichrome. Scale bar: **A**,**C**,**D**: 10 mm; **B**,**D**,**F** 235 μm.

To evaluate the in vivo properties of FS1-1× cultured for 4 and 10 days at the A/L, they were grafted on athymic mice and compared with FS3-1× cultured for 10 days at the A/L as control. Twenty-one days after grafting, FS1-1× cultured for 4 days at the A/L showed a similar overall macroscopic aspect when compared with FS3-1× cultured for 10 days at the A/L (Figure 2A,E). The presence of a fully differentiated epidermis well attached to the underlying dermis was confirmed by histological analyses (Figure 2B,F, arrows). This result indicates that TES cultured for 4 days at the A/L progresses into a mature skin tissue after grafting. However, the macroscopic aspect of FS1-1× cultured for 10 days at the A/L (Figure 2C) was less homogeneous compared with control TES (Figure 2E). Histological analysis showed absence of epidermis in several areas (Figure 2D). Given that FS1-1× shelf-life is relatively short and does not satisfy the needs of stability overtime, the study of FS1-1× was not further pursued. 

**Figure 3:**
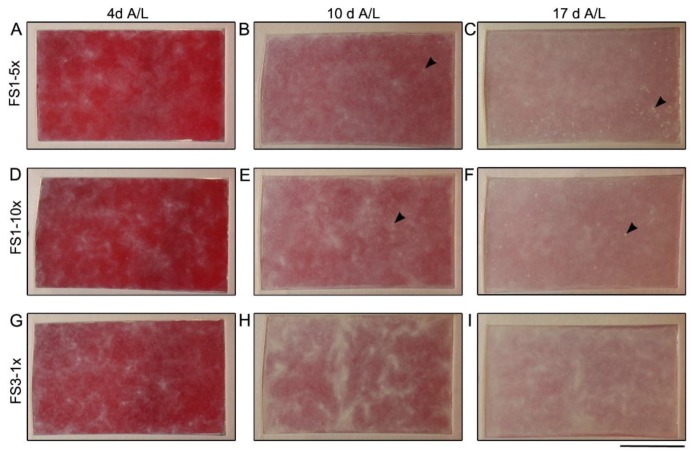
Macroscopic analysis of tissue-engineered skin substitutes matured in vitro. Top view pictures of FS1-5× (**A–C**), FS1-10× (**D–F**), and FS3-1× (**G–I**) cultured for 4 (4 A/L), 10 (10 A/L), and 17 (17 A/L) days at the air–liquid interface. Arrowheads point opaque dots correlated with keratinocyte infiltrations across and under the reconstructed dermis. The red appearance is due to the culture media (red) that is under the TES. The culture media (red) under the FS1 is more apparent when the stratum corneum of the epidermis is thinner. Scale bar: 25 mm.

To circumvent the problem of ECM digestion observed in FS1-1× cultured for 10 days at the A/L, and to increase stability overtime, the fibroblast seeding density was increased 5 (5×) and 10 (10×) times. The resulting TESs are herein after referred to as FS1-5× and FS1-10×, respectively. Tissues were evaluated after 4, 10, and 17 days of culture at the A/L. In contrast with FS1-1× (Figure 1D), no clear zone appeared overtime on the surface of FS1-5× and FS1-10× (Figure 3A–C and Figure 3D–F, respectively). Slightly different opaqueness was noted after 4 and 10 days of culture at the A/L. However, this difference was not visible when the culture was continued to 17 days at the A/L; all TESs presented homogenous opacity (Figure 3C,F,I). Small opaque dots, previously correlated to keratinocyte infiltration forming an epithelial inclusion within the reconstructed dermis, were observed after 10 days of culture at the A/L in both, FS1-5× and FS1-10× (Figure 3B and Figure 3E, respectively, arrowheads). They were in larger number in FS1-5× where the mean (±standard deviation) was 117.3 ± 49.0 (n = 3 [sample size], N = 1 [population size]) compared with 51.3 ± 12.7 (n = 3, N = 1) for FS1-10×. After 17 days of culture at the A/L, the mean number of opaque dots remained stable in FS1-5× (111.0 ± 7.5 [n = 3, N = 1]) and FS1-10× (52.5 ± 14.8 [n = 3, N = 1]) but grew larger (Figure 3C,F, respectively, arrowheads). 

**Figure 4:**
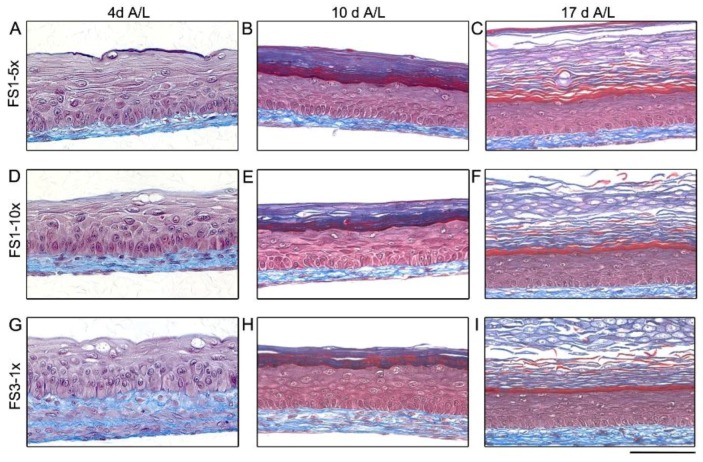
Histological analysis of tissue-engineered skin substitutes matured in vitro. Representative histological results of TES produced with FS1-5× (**A–C**), FS1-10× (**D–F**), and FS3-1× (**G–I**) cultured for 4 (4 A/L), 10 (10 A/L) and 17 (17 A/L) days at the air–liquid interface. Histological coloration: Masson’s trichrome. Scale bar: 100 µm.

After 4 days of culture at the A/L, histological features of TES produced with FS1-5× (Figure 4A) and FS1-10× (Figure 4D) tissues were similar to FS3-1× (Figure 4G). For both FS1-5× and FS1-10×, the reconstructed dermis appeared thinner compared with FS3-1×, but the ECM network looked denser, especially for FS1-10× (Figure 4D). No important ECM digestion zone was observed within the reconstructed dermis. After 10 and 17 days of culture at the A/L, the presence of a fully differentiated epidermis presenting all 4 layers of normal human epidermis (stratum basale, spinosum, granulosum, and corneum) was observed in FS1-5× (Figure 4B,C) and FS1-10× (Figure 4E,F) and was similar to FS3-1× in both cases (Figure 4H,I). Again, keratinocyte inclusions corresponding to opaque dots observed macroscopically were noted in the dermis of FS1-5× and FS1-10× (data not shown).

### 2.2. Immunofluorescence Analysis 

**Figure 5:**
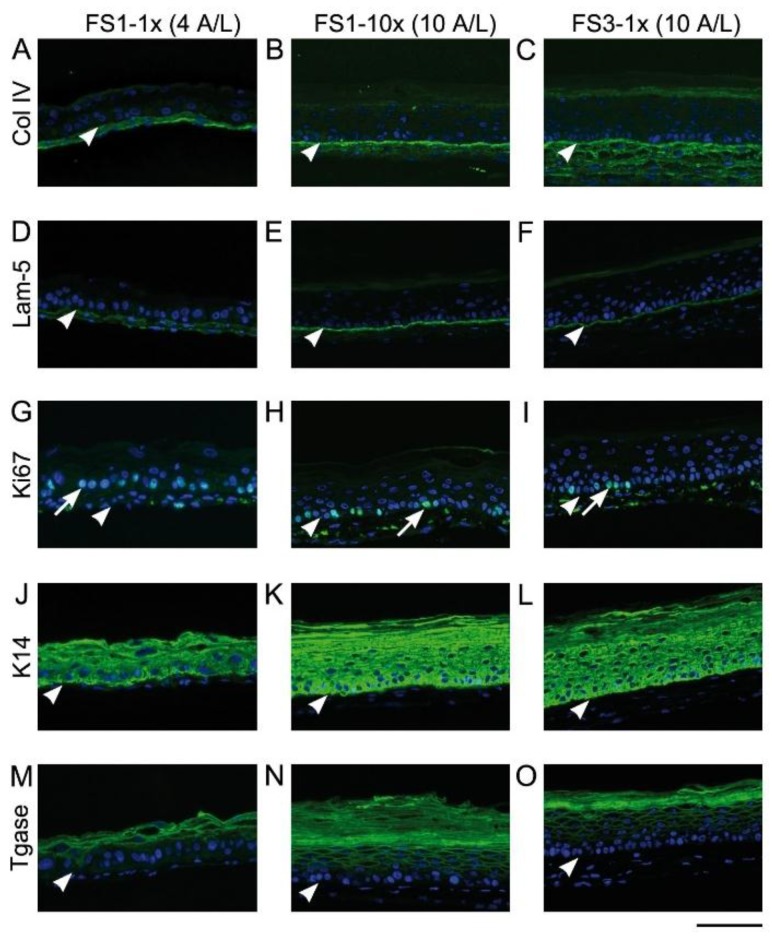
Analysis of skin marker expression in tissue-engineered skin substitutes matured in vitro. Representative pictures FS1-1× (left panel), FS1-10× (center panel), and FS3-1× (right panel) cultured for 4 (4 A/L) and 10 (10 A/L) days at the air–liquid interface immunolabeled for the detection of type IV collagen (**A**–**C**), laminin 5 (**D–F**), Ki67 (**G**–**I**), keratin 14 (**J**–**L**), and transglutaminase (**M**–**O**). Arrowheads point out the level of the dermo-epidermal junction. Arrows point out nuclei expressing the proliferation marker Ki67. Col IV, type IV collagen; K14, keratin 14; Lam-5, laminin 5; Tgase, transglutaminase. Scale bar: 100 µm.

Figure 5 shows the expression of different skin markers in TESs cultured for 4 and 10 days at the A/L. Regardless of the fibroblast seeding density and the number of fibroblast-derived tissue sheets used, after 4 days of culture at the A/L, a discontinuous diffuse labeling of type IV collagen and laminin 5 was observed at the dermo-epidermal junction of TESs (Figure 5A,D, respectively) while the labeling was continuous and concentrated at the dermo-epidermal junction after 10 (Figure 5B,E) and 17 (Figure 5C,F) days. Keratinocyte proliferation marker Ki67 was detected in the nucleus of many cells within the stratum basale of all tested TESs (Figure 5G–I). As expected, keratin (K) 14 was detected within stratum basale (Figure 5J–L) and K10 within suprabasal layers (data not shown) of all TES epidermis. Transglutaminase was detected at day 4 of culture at the A/L (Figure 5M), and expression extended over more suprabasal layers at days 10 (Figure 5N,O) and 17 (data not shown).

### 2.3. Tissue Contraction 

**Figure 6:**
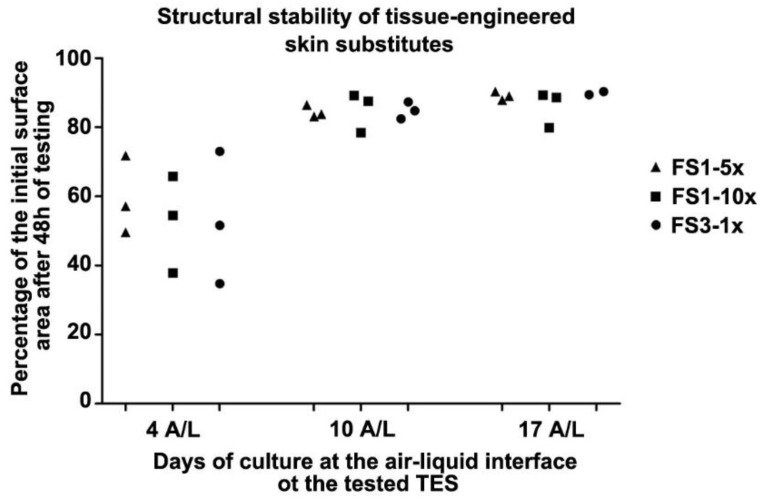
Structural stability of tissue-engineered skin substitutes matured in vitro for various time at the air–liquid interface before measuring surface area after an additional 48 h on agar substrate. Resulting contraction indicated as a percentage of the initial (at t = 0 h on agar) surface area of TES FS1-5× 10d A/L (triangles), FS1-10× (squares), and FS3-1× (circles) cultured for 4 (4 A/L), 10 (10 A/L), and 17 (17 A/L) days at the air–liquid interface remaining after 48 h of in vitro testing on agar.

The TES surface available for the graft or wound dressing depends on the contractility of the tissue after being detached from the paper frame. To evaluate the structural stability of TES, FS1-5×, FS1-10×, and FS3-1× were placed on agar substrates and the surface area of each skin substitute was measured over time to obtain contraction kinetic curves. As observed in a previous study with self-assembled skin substitutes [31], most of the contraction occurred within two hours, and the contraction curves reached equilibrium thereafter (data not shown). After 48 h, the final contraction of tissues cultured for 4 days at the A/L was higher than tissues cultured for 10 and 17 days (Figure 6). No association between fibroblast seeding density, number of stacked fibroblast sheets, and final contraction was observed.

## 3. Discussion

In this study, we proposed an adaptation of the scaffold-free cell-based method of self-assembly allowing for a 25-day production of living dermo-epidermal skin substitutes which retain their histological characteristics in vitro for the two week period studied. Our work highlights the importance of the fibroblast seeding density in obtaining a FS that can support keratinocyte culture and limits ECM degradation. In comparison with the previously reported methods to produce SASS [28,31], the procedure proposed here requires fewer laboratory manipulations. The resulting TES could be manufactured using donor cells from biobanks allowing standardizing procedures for increased productivity and efficiency.

Weekly application of bilayered skin substitutes containing living fibroblasts and keratinocytes is known as favouring definitive closure of ulcers that have been unresponsive to conventional therapies [11]. The TES presented here was designed to serve as a temporary biological dressing to stimulate healing of hard-to-heal ulcers, such as ulcers of large size or which have lasted for more than one year [6,7,8]. In the context of a temporary use, the expected wound healing promoted by our TES model may be comparable to other skin substitutes composed of living fibroblasts and keratinocytes [11,16,18,30,32,33,34]. Cells composing such dressings are capable of adapting and responding to the wound environment; an epidermal barrier is formed and interaction between cells results in the secretion of growth factors, cytokines, and ECM material that can foster healing.

The ability of human fibroblasts to secrete ECM varies between donors [28]. In the present study, the fibroblast populations used to produce TESs were selected on the basis of their high ECM production capacities. Nevertheless, after the addition of keratinocytes, ECM digestion signs were observed in some TESs produced with a single FS. This phenomenon can be explained by the imbalance between ECM and matrix metalloproteinases (MMPs). Indeed, in collagen-based skin substitutes, important MMP activity has been observed in the dermis after the addition of keratinocytes [13,35]. In the present study, we showed that with the self-assembly method, the problem of ECM digestion is manageable by stacking more than one FS, or by seeding fibroblasts at a higher density. Increasing ECM density, reseeding fibroblasts [36], or adding agents that limit collagen degradation, such as aprotinin or MMP inhibitors [13,37,38], would be additional alternative options, but they would all require supplementary handling and management. 

To optimize the biologically active surface area, structural stability (or limited contraction of the final substitute) is another aspect to consider when developing skin substitutes. In accordance with previous work showing the importance of epidermal differentiation for the stabilization of TES, TES cultured for 4 days at the A/L were more contractile (loss of about 45% of initial surface area) compared with TES cultured for 10 days at the A/L where TES reached structural stability (loss of only 14% of the initial surface area in average). Nevertheless, the amount of contraction experienced in TES cultured for 4 days at the A/L after frame detachment is not problematic for a temporary dressing which role is to provide wound healing soluble factors by keratinocyte–fibroblast interactions [12,14]. The progression of TES cultured for 4 days at the A/L into a mature skin after grafting on athymic mice also suggested that a fully differentiated epithelium before grafting may not be necessary to stimulate wound healing. 

To accelerate healing of large ulcers, TES containing autologous keratinocytes may be advantageous because keratinocytes could incorporate into the wound bed and participate in the epithelialization. However, for permanent integration of the graft into the host, keratinocytes have to be autologous because they express high levels of markers responsible for acute rejection [39,40]. For a more personalized treatment, a unique FS, using the self-assembly method, would be possible for the production of bilayered skin substitute containing autologous keratinocytes. However, taking into account that ECM secretion yields vary between individuals, this method may be difficult—although not impossible—to manage with autologous fibroblasts. Some evidences show that an ECM scaffold containing living allogenic fibroblasts does not create adverse host responses in vivo [16,41,42,43]. Therefore, for a temporary use, TES produced with an allogenic human fibroblast population selected for its biological qualities and biosafety, complemented with autologous keratinocytes, could be a valuable option. 

## 4. Materials and Methods 

### 4.1. Cell Populations

This study was conducted in accordance with the Declaration of Helsinki and the protocols were approved by the institution’s animal care committee (comité de protection des animaux de laboratoire de l’Université Laval, Québec, Canada) and by the institution’s committee for the protection of human subjects (comité d’éthique de la recherche du Centre de recherche du CHU de Québec-Université Laval).

### 4.2. Cell Isolation and Culture

Human keratinocytes and dermal fibroblasts were isolated as previously described [44] from skin biopsies originating from breast surgery of 3 healthy women (26-year-old for keratinocytes and 18- and 38-year-old for fibroblasts). Dermal fibroblasts were cultured in Dulbecco–Vogt modified Eagle medium (DMEM, Corning, Corning, NY, USA) supplemented with 10% fetal bovine serum (FBS, Seradigm, Providence, UT, USA) and antibiotics (100 U/mL penicillin [Sigma-Aldrich, Saint-Louis, MO, USA], and 25 μg/mL gentamicin [Schering, Pointe-Claire, QC, Canada]). Keratinocytes were cultured on a lethally irradiated human fibroblast feeder layer [45] in DMEM with Ham’s F12 at a ratio 3:1 (DMEM-Ham; Corning, Corning, NY, USA) supplemented with 5% Fetal Clone II serum (GE Healthcare, Chicago, IL, USA), and containing 5 μg/mL insulin (Sigma-Aldrich, Saint-Louis, MO, USA), 0.4 μg/mL hydrocortisone (Calbiochem, La Jolla, CA, USA), 0.2 μg/mL isoproterenol hydrochloride (Sandoz, Québec, QC, Canada), 10 ng/mL epidermal growth factor (Austral Biological, San Ramon, CA, USA) and antibiotics. Both cell types were maintained at 37 °C in a humidified incubator containing 8% carbon dioxide (CO_2_) and medium was changed 3 times a week. For tissue production, fibroblasts were used at passage 5 and 6 and keratinocytes at passage 2.

### 4.3. Tissue-Engineered Skin Production

The TES production methods presented here originate from the method previously described [28] and referred to as SASS-3. Fibroblasts were seeded at a density of 4 × 10^3^ cells/cm^2^ (1×), 2 × 10^4^ cells/cm^2^ (5×), or 4 × 10^4^ cells/cm^2^ (10×) in 85 cm^2^ Nunc™ Omnitray™ tissue culture plates with removable lids (Thermo Fischer Scientific, Rochester, NY, USA) and cultured in fibroblast medium containing 50 μg/mL of ascorbic acid to stimulate ECM synthesis. After 17 days, 1 × 10^5^ keratinocytes per cm^2^ were directly added on FS still attached to the bottom of the culture plate. After 4 days of submerged culture in keratinocyte medium supplemented with 50 μg/mL ascorbic acid (Galenova Inc., St-Hyacinthe, QC, Canada), a custom made filter paper frame was placed onto the tissue constructs. The paper frame being slightly smaller than the culture flask allowed for the surrounding tissue to be folded on top of the paper frame. By grasping the frame with forceps, constructs were carefully detached from the culture plates and were either stacked or not (FS1) with a FS cultured 21-day. In the latter case, the surrounding tissue was again folded on top of the frame, the constructs were then detached from the culture plates and the procedure was repeated with another FS. These resulting constructs (FS3) were secured on their frames with LigaClips (Ethicon Endo-Surgery, Cincinnati, OH, USA). Each construct (FS1 and FS3) was then transferred onto a polypropylene membrane (Spectra/Mesh^®^Woven polypropylene membrane filters, Fisher Scientific, Ottawa, ON, Canada) laying on custom-made acrylic support to hold the tissues at the A/L interface. Tissues were further cultured for 4, 10, or 17 days at the A/L in keratinocyte medium containing 50 μg/mL ascorbic acid and exempt of epidermal growth factor. Each proposed method was performed two times (with fibroblasts from two different donors), each time in triplicate.

### 4.4. Contraction Kinetics on Agar Substrate

In order to assess structural stability of the substitutes, measurements were performed to evaluate contraction of TES previously cultured for 4, 10, and 17 days at the A/L interface according to the in vitro method previously described [31]. Succinctly, an Adaptic™ non-adherent sterile gauze (Acelity, San Antonio, TX, USA) was placed on the top of each TES, which was then transferred onto a sterile cutting board. A 30 mm × 30 mm square tissue sample was cut off using a custom-made stainless steel die-cutter and immediately transferred on a soft gel substrate (1% agar (Sigma-Aldrich, Saint-Louis, MO, USA) in DMEM (Corning, Corning, NY, USA)). A millimetric grid was positioned on the agar substrate, beside the TES as size reference. To evaluate tissue contraction over time, pictures were taken after 48 h. Substitutes were manipulated under sterile conditions and were maintained in the incubator between photo shootings. TES surface area was measured using ImageJ^®^ software (NIH, Bethesda, MD, USA). The contractions were expressed as percentage of the initial value (9 cm^2^ surface area obtained with the die-cutter).

### 4.5. Grafting on Athymic Mice

Athymic male nu/nu mice (Charles River Laboratories, Lasalle, QC, Canada) were used for tissue grafting and maintained under sterile housing. To prevent infections, they received Ceftazidime antibiotic (3 mg/mouse, GlaxoSmithKline Inc., Mississauga, ON, Canada) 48 and 24 h before surgery and preoperatively. To provide analgesia, buprenorphine (0.05–0.1 mg/kg, Champion Alstoe, Whitby, ON, Canada) was administered before surgery, as well as daily doses of Caprofen (5–10 mg/kg, Pfizer, Kirkland, QC, Canada) for 48 h post-operation. Grafting was performed as previously described [46]. Briefly, reconstructed TES were detached from their paper frame. A 2.5 cm × 2.5 cm square portion of each TES was removed and a sterile Adaptic™ non-adherent gauze (Acelity, San Antonio, TX, USA) was positioned on the top to facilitate transport. Each TES was applied on the mid-back of each animal, onto the muscular bed, and the gauze was removed. After the grafting procedure, mice were inspected regularly for signs of infections and necrosis and pictures were taken at days 0, 4, and 21 to track graft integration. Mice were sacrificed on day 21, and TES biopsies were taken and processed for histological and immunofluorescence analysis. Each condition was grafted in triplicate. 

### 4.6. Histological and Immunofluorescence Analyses

For histological analyses, samples were fixed overnight in Histochoice (Amresco, Solon, OH, USA) and embedded in paraffin. Five-micrometer thick sections were stained with Masson’s Trichrome using Weigert’s hematoxylin, fuchsin-ponceau, and aniline blue.

For immunofluorescence analyses, samples were embedded in Tissue-Tek OCT compound (Sakura Finetek, Torrance, CA, USA) and frozen in liquid nitrogen. Indirect immunofluorescence assays were performed on 5 μm-thick cryosections permeabilized with acetone (10 min at −20 °C). The antibodies used were: rabbit polyclonal anti-human keratin (K) 14 (Cedarlane, Burlington, ON, Canada), anti-human type IV collagen (Abcam, Toronto, ON, Canada), anti-human ki67 (Abcam, Toronto, ON, Canada), anti-human transglutaminase 1 (TGM1) (Proteintech Group Inc. Rosemont, IL, USA) and anti-human laminin (Abcam, Toronto, ON, Canada). Negative controls consisted in the omission of primary antibodies during labeling reaction and normal human skin was used as positive control. As secondary antibody Alexa-488-conjugated donkey anti-rabbit (Life technologies, Carlsbad, CA, USA) was used. Cell nuclei were counterstained with Hoechst reagent 33258 (Sigma-Aldrich, Saint-Louis, MO, USA). 

Histological and immunofluorescence sections were observed under Zeiss Axio Imager (Carl Zeiss Canada Ltd, Toronto, ON, Canada) and photographed with AxioCam ICc1digital camera. Images were processed using and AxioVision 4.8.2 software (Carl Zeiss Canada Ltd, Toronto, ON, Canada) and Adobe Photoshop CS4 (Adobe System, San Jose, CA, USA).

## 5. Conclusions

In conclusion, our alternative production method of TES using a unique FS can be used for the production of bilayered skin substitutes. Increasing the fibroblast seeding density 5 and 10 times allowed the production of a conjunctive tissue supporting a fully differentiated epithelium for the entire period studied (2 weeks). The produced TESs have also demonstrated similar histological aspects, protein secretion, and contractility with control tissues, for which clinical utility in wound healing of patients has been reported [29,30].
